# Gallbladder adenomyomatosis: imaging findings, tricks and pitfalls

**DOI:** 10.1007/s13244-017-0544-7

**Published:** 2017-01-26

**Authors:** Matteo Bonatti, Norberto Vezzali, Fabio Lombardo, Federica Ferro, Giulia Zamboni, Martina Tauber, Giampietro Bonatti

**Affiliations:** 1 Department of Radiology, Bolzano Central Hospital, 5 Boehler Street, 39100 Bolzano, Italy; 2 Department of Radiology, University of Verona, 10 LA Scuro Place, 37134 Verona, Italy; 3 Department of Pathology, Bolzano Central Hospital, 5 Boehler Street, 39100 Bolzano, Italy

**Keywords:** Gallbladder, Rokitansky–Aschoff sinuses of the gallbladder, Gallbladder diseases, Ultrasonography, Magnetic resonance imaging

## Abstract

**Abstract:**

Gallbladder adenomyomatosis (GA) is a benign alteration of the gallbladder wall that can be found in up to 9% of patients. GA is characterized by a gallbladder wall thickening containing small bile-filled cystic spaces (i.e., the Rokitansky–Aschoff sinuses, RAS). The bile contained in RAS may undergo a progressive concentration process leading to crystal precipitation and calcification development. A correct characterization of GA is fundamental in order to avoid unnecessary cholecystectomies. Ultrasound (US) is the imaging modality of choice for diagnosing GA; the use of high-frequency probes and a precise focal depth adjustment enable correct identification and characterization of GA in the majority of cases. Contrast-enhanced ultrasound (CEUS) can be performed if RAS cannot be clearly identified at baseline US: RAS appear avascular at CEUS, independently from their content. Magnetic resonance imaging (MRI) should be reserved for cases that are unclear on US and CEUS. At MRI, RAS can be identified with extremely high sensitivity, but their signal intensity varies widely according to their content. Positron emission tomography (PET) may be helpful for excluding malignancy in selected cases. Computed tomography (CT) and cholangiography are not routinely indicated in the suspicion of GA.

**Teaching points:**

1. Gallbladder adenomyomatosis is a common benign lesion (1–9% of the patients).

2. Identification of Rokitansky–Aschoff sinuses is crucial for diagnosing gallbladder adenomyomatosis.

3. Sonography is the imaging modality of choice for diagnosing gallbladder adenomyomatosis.

4. Intravenous contrast material administration increases ultrasound accuracy in diagnosing gallbladder adenomyomatosis.

5. Magnetic resonance is a problem-solving technique for unclear cases.

## Main text

### Gallbladder adenomyomatosis

**Fig. 1:**
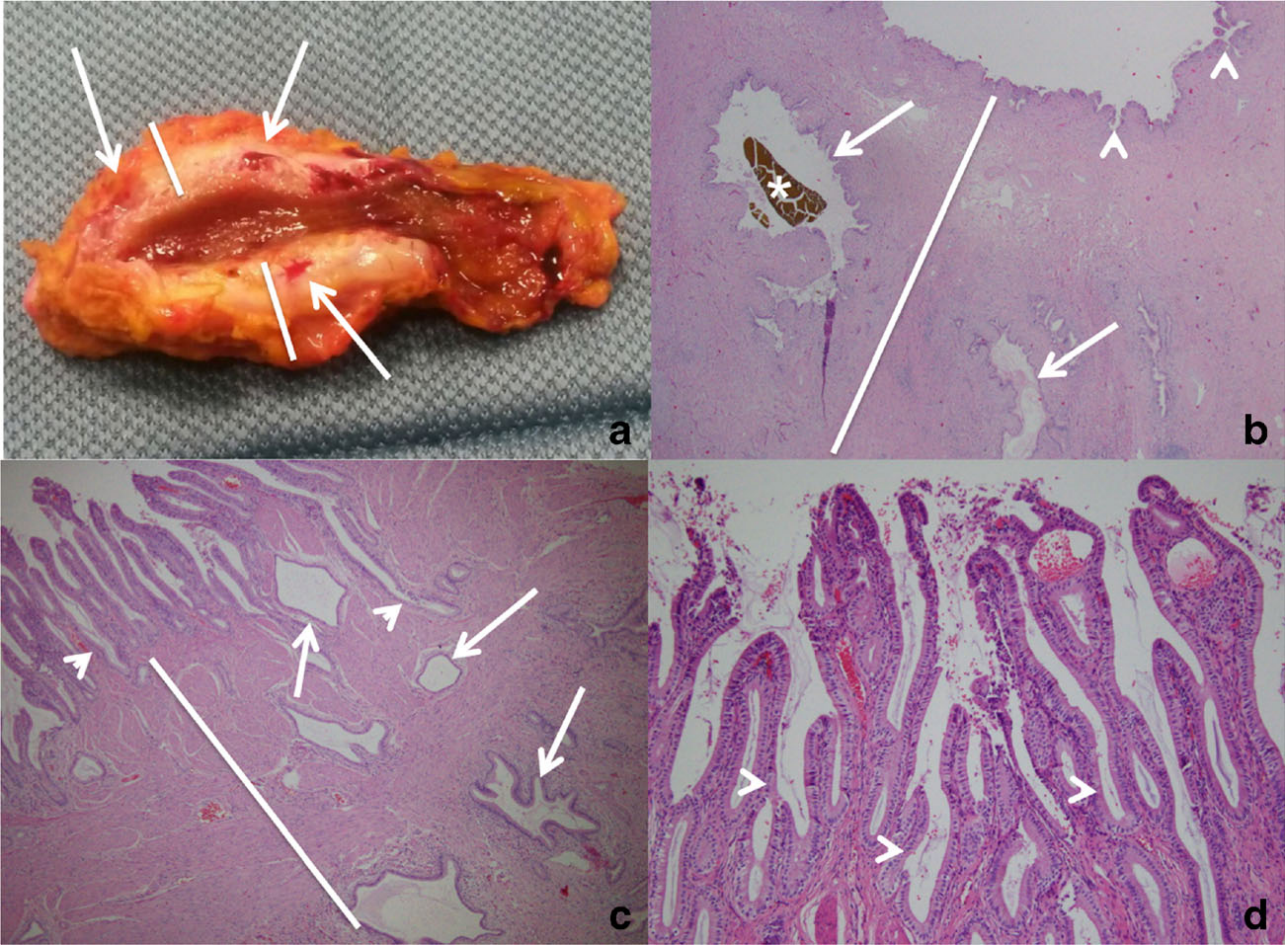
Gallbladder adenomyomatosis: pathology findings. Macroscopically (**a**) GA is characterized by gallbladder wall thickening (*lines*) containing small cystic spaces (arrows) representing Rokitansky–Aschoff sinuses. Microscopically, at low (2×) magnification (**b** and **c**), wall thickening is due to hyperplasia of the muscular layer (*lines*); a variable degree of epithelial proliferation (*arrowheads*) is also appreciable and epithelium-lined cystic spaces, representing RAS (*arrows*), can be observed within the muscular layer. Biliary stones (*star*) may be present within RAS. At high (40×) magnification (**d**), the proliferative mucosal glandular component that leads to epithelial infolding (*arrowheads*) and RAS formation is better recognizable

Gallbladder adenomyomatosis (GA) is a benign alteration of the gallbladder wall characterized by excessive epithelial proliferation associated with hyperplasia of the muscularis propria, resulting in gallbladder wall thickening. The excessive epithelial proliferation leads to epithelial infolding within the underlying muscular layer with subsequent formation of epithelium-lined diverticular pouches, the so-called Rokitansky–Aschoff sinuses (RAS; Fig. 1) [1–4]. The content of RAS consists of bile that may undergo progressive dehydration over time, leading to cholesterine crystal precipitation [5]. Moreover, cholesterine crystals may induce a chronic inflammatory reaction leading to intramural dystrophic calcification development. The serosa is never involved by GA.

**Fig. 2:**
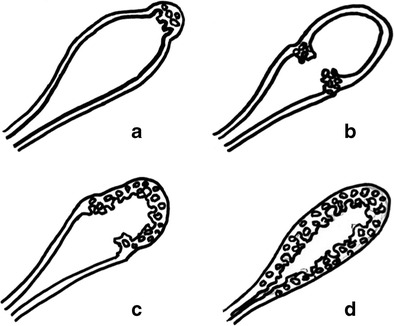
Gallbladder adenomyomatosis: patterns of gallbladder wall involvement. Drawings showing localized gallbladder adenomyomatosis (**a**), annular gallbladder adenomyomatosis (**b**), segmental gallbladder adenomyomatosis (**c**) and diffuse gallbladder adenomyomatosis (**d**)

Adenomyomatosis may involve the gallbladder according to four main patterns: localized, segmental, annular and diffuse (Fig. 2) [6, 7].

*Localized GA* is the most common pattern and is characterized by a focal thickening, usually involving the fundal region (the so-called “*fundal GA*”). The uninvolved gallbladder wall appears physiologically thin and the overall gallbladder shape is usually maintained.

*Segmental GA* is characterized by the involvement of a larger portion of the gallbladder wall, typically the fundus and the distal third of the body. The involved portion appears contracted, whereas the uninvolved one maintains its normal shape.

*Annular GA* is characterized by a ring-form thickening of the gallbladder wall, usually involving the middle portion. The gallbladder appears contracted only in the involved portion, changing its global morphology and becoming “hourglass-shaped”. In some cases, epithelial proliferation may be particularly conspicuous and subdivide the gallbladder lumen into two separate compartments. As a consequence, biliary sludge and stones may accumulate into the isolated fundal compartment [8]. According to some authors, annular GA should be considered a subtype of segmental GA [9].

*Diffuse GA* is characterized by the involvement of the whole organ that consequently appears contracted, even after fasting.

The pathogenesis of GA is not fully understood: an association with gallbladder stones and chronic inflammatory changes has been highlighted in many studies [1, 5, 10–13], but a correlation with acquired wall motility as a consequence of increased endoluminal pressure has also been postulated [14, 15]. GA is a benign lesion as the hyperplastic epithelium of GA has no higher neoplastic potential than that of a normal gallbladder, even though gallbladder carcinoma may also arise in association with GA [16]. Some studies have shown an increase in gallbladder cancer prevalence among patients with segmental type adenomyomatosis compared to patients without GA or with other patterns of GA, in particular, in the elderly. However, these results may have been influenced by the higher prevalence of cholecystolithiasis in patients affected by segmental type GA, which represents a well-known risk factor for gallbladder carcinoma [10–13, 16]. GA may increase in size over time and this change by itself must not be considered an index of malignancy [17]. Patients affected by GA are usually asymptomatic. When present, symptoms may include right upper quadrant pain, possibly also as a consequence of the presence of gallbladder stones.

GA is frequently observed in cholecystectomy specimens, with a reported prevalence of 1–9% in pathology series [1, 4, 9, 13]. GA represents about 40% of benign gallbladder lesions [1, 4].

**Fig. 3:**
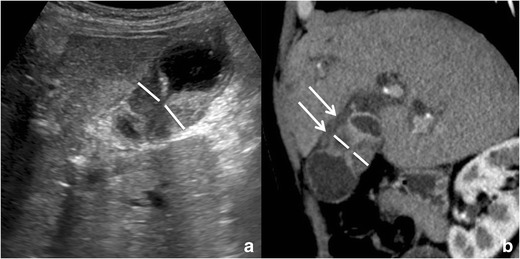
Gallbladder cancer: gallbladder adenocarcinoma may involve the gallbladder wall with various patterns. This case of gallbladder adenocarcinoma with annular involvement (*white lines*) can be differentiated from an adenomyomatosis because of the absence of cystic spaces (RAS) within the wall thickening on ultrasound (**a**) and because of the presence of hypodense tissue between the gallbladder wall and the adjacent liver (*arrows*) on contrast-enhanced CT (**b**)

Given its relatively high prevalence and the continuous increase in imaging studies performance, GA can be frequently encountered during everyday practice. The radiologist plays a central role in the diagnosis of GA and its main aim is to distinguish GA from neoplastic gallbladder wall thickenings (Fig. 3) in order to avoid unnecessary cholecystectomies. It is also important to accurately describe the gallbladder wall involvement pattern, as it can modify patient management.

There are no universally accepted guidelines for GA management. Given the lack of malignancy potential, GA is usually considered a “don’t touch” lesion and cholecystectomy should be routinely reserved for symptomatic patients only or in case of inconclusive imaging findings. In any case, the surgical option might be considered in patients with segmental type GA, given its higher association with gallbladder cancer, and in patients with diffuse GA, given the possible difficulties in identifying neoplastic foci within the wall thickening [18].

In this article, we review multimodality imaging findings of GA, providing tips that may increase diagnostic confidence and highlighting possible pitfalls.

## Imaging of gallbladder adenomyomatosis

Besides GA, differential diagnosis of gallbladder wall thickenings includes the post-prandial state, acute and chronic cholecystitis, cholesterine polyps, neoplasms and many other less common conditions. Independently from the radiological modality, an imaging clue for diagnosing GA is the detection, within a thickened gallbladder wall, of Rokitansky–Aschoff sinuses. It must be kept in mind that RAS may show extremely different imaging features according to their variable content that may range from clear bile to calcifications. Moreover, it must be considered that tiny cystic spaces, resembling RAS, have been identified also in rare cases of mucine-producing gallbladder carcinomas [19]; anyway, overall lesion shape in these neoplasms was much more irregular than in cases of GA.

## Oral cholecystography

**Fig. 4:**
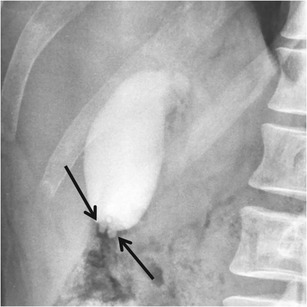
Gallbladder adenomyomatosis: typical oral cholecystography findings. In this case of fundal type GA, RAS (*arrows*) are filled by contrast material as a consequence of their communication with the gallbladder lumen. Courtesy of Marco Ferigato, radiographer at Bolzano Central Hospital

Oral cholecystography (OC) was the first imaging modality used for diagnosing GA, but nowadays represents an obsolete technique. The knowledge of typical GA findings at OC, however, enables better understanding of the imaging patterns we are now dealing with. In particular, besides gallbladder wall thickening and possible strictures formation, the most relevant finding at OC was the visualization of rounded contrast media collections adjacent to the gallbladder lumen, representing RAS (Fig. 4) [20]. This finding represents the imaging demonstration of the communication between RAS and the gallbladder lumen.

## Ultrasound

Trans-abdominal ultrasound (US) represents the imaging modality of choice for the detection and characterization of GA, with accuracy values that range from 91.5 to 94.8% in differentiating GA from early-stage gallbladder cancer [21].

### Imaging findings

**Fig. 5:**
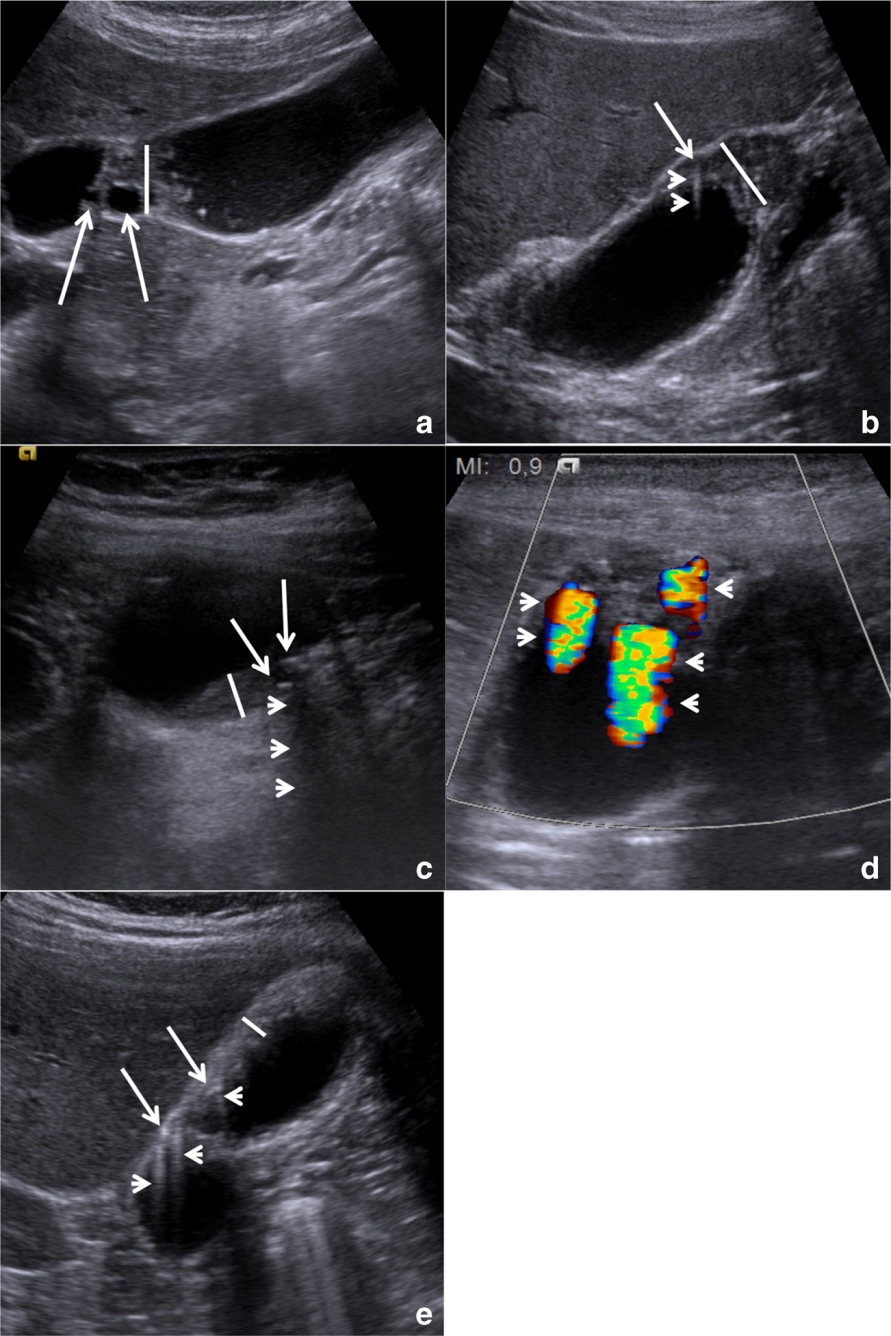
Gallbladder adenomyomatosis: typical US findings in annular type (**a**), fundal type (**b** and **d**), segmental type (**c**) and diffuse type (**e**). Gallbladder wall thickening (*line*) is always seen in gallbladder adenomyomatosis, but it is non-specific. On b-mode images, Rokitansky–Aschoff sinuses (*arrows*) typically appear anechoic (**a**), but they can also appear hyperechoic if cholesterine crystals or calcifications are present (**b** and **c**). Comet-tail reverberation artefacts (Figures *b* and *e*, *arrowheads*) or acoustic shadowing (**c**, *arrowheads*) are usually observed profoundly in RAS. On colour Doppler images (**d**), twinkling artefacts (*arrowheads*) may be observed profoundly in RAS

Focal or diffuse *gallbladder wall thickening* (Fig. 5) can be easily detected at US and represents the consequence of both epithelial hypertrophy and muscular hyperplasia. Wall thickening represents a hallmark of GA, being always present, but it is poorly specific, as it can be found in most gallbladder pathologies. Anyway, in GA, the outer gallbladder layer must appear sharp and a clear cleavage plane with the liver must always be present. No pericholecystic fluid should be observed.

Small *anechoic cystic spaces* (1 – 10 mm) representing clear bile-filled RAS should be recognized within the thickened gallbladder, being pathognomonic for GA (Fig. 5a). Whenever cholesterine crystals fill RAS, they appear as intramural *echogenic spots* in association with reverberation artefacts (Fig. 5b and e). Reverberation artefacts are the consequence of the coexistence of different acoustic impedance media, i.e., clear bile and cholesterine crystals, within RAS and appear as hyperechoic *“comet-tail” artefacts* that project deeply into RAS. Sometimes RAS themselves may be not directly recognizable at the origin of reverberation artefacts. Calcification-filled RAS appear as intramural *echogenic spots associated with* posterior acoustic shadowing (Fig. 5c). Also, the presence of cholesterine crystal- or calcification-filled RAS is virtually diagnostic for GA.

*Twinkling artefacts* on colour Doppler ultrasound (Fig. 5d) are due to the interaction of the ultrasound beam with a rough acoustic interface composed by randomly disposed strongly reflecting media (i.e., cholesterine crystals or calcifications) [22]. Twinkling artefacts appear as rapidly alternating red and blue colour Doppler signals, “comet-tail” shaped, deeply in RAS, and are better appreciable using low-frequency probes [23]. Their presence is strongly associated with GA.

### Tips & tricks

Patient’s *fasting* is fundamental in order to correctly evaluate the gallbladder and, in particular, whenever dealing with gallbladder wall thickenings. A minimum of 8 h of fasting is recommended before upper abdomen sonography.

A precise *focal depth adjustment* is crucial in order to correctly investigate every portion of the gallbladder wall for the presence of GA. In particular, it is often necessary to set the focal point to a very superficial position in order to evaluate patients with fundal type GA.

**Fig. 6:**
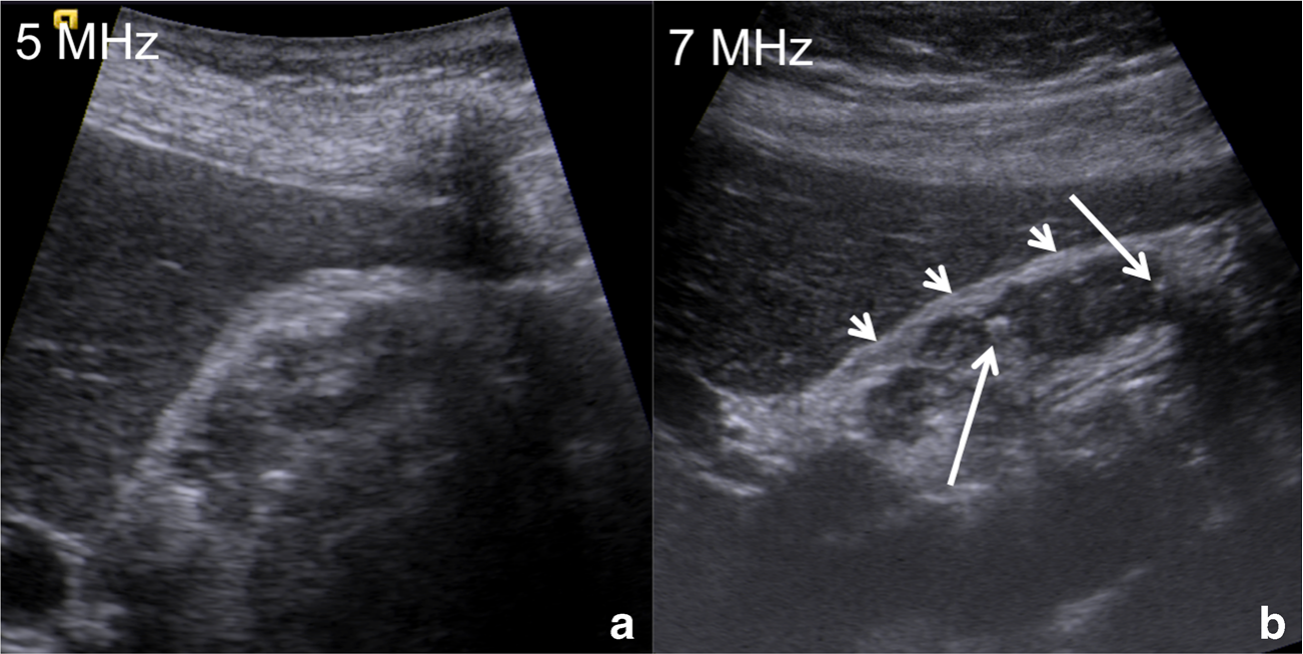
US of gallbladder adenomyomatosis: use of different frequencies probes. In this patient with diffuse GA, the gallbladder wall is poorly evaluable by means of a conventional 5-MHz convex probe (**a**). Using a high-resolution 7-MHz linear probe (**b**) hyperechoic Rokitansky–Aschoff sinuses (*arrows*) can be highlighted within a diffusely thickened gallbladder wall; moreover, the serosa maintains sharp margins (*arrowheads*)

The use of *high-frequency probes* (Fig. 6) increases US accuracy in the diagnosis of GA. Indeed, GA often involves gallbladder fundus, which is usually unsatisfactorily evaluated by means of the classical 4–5-MHz convex probes; every suspicious finding in this area must be further investigated by means of higher frequency (7–9 MHz) linear probes for better characterization.

The introduction of *harmonic imaging* has increased US accuracy in depiction of gallbladder wall morphology and in detection of Rokitansky–Aschoff sinuses. Harmonic imaging should always be used in the suspicion of GA.

GA is sometimes poorly visible with the classical sub-costal approach, particularly in obese patients. The interposition of hepatic parenchyma between the probe and the gallbladder wall (i.e., the so-called *hepatic window*) may overcome this limitation, increasing image quality.

### Pitfalls & limitations

US is an *operator-dependent* imaging modality and the ability in depicting GA varies according to the operator’s experience. Moreover, sonographic examination may be limited in case of *obese* patients and bowel *gas interposition*.

**Fig. 7:**
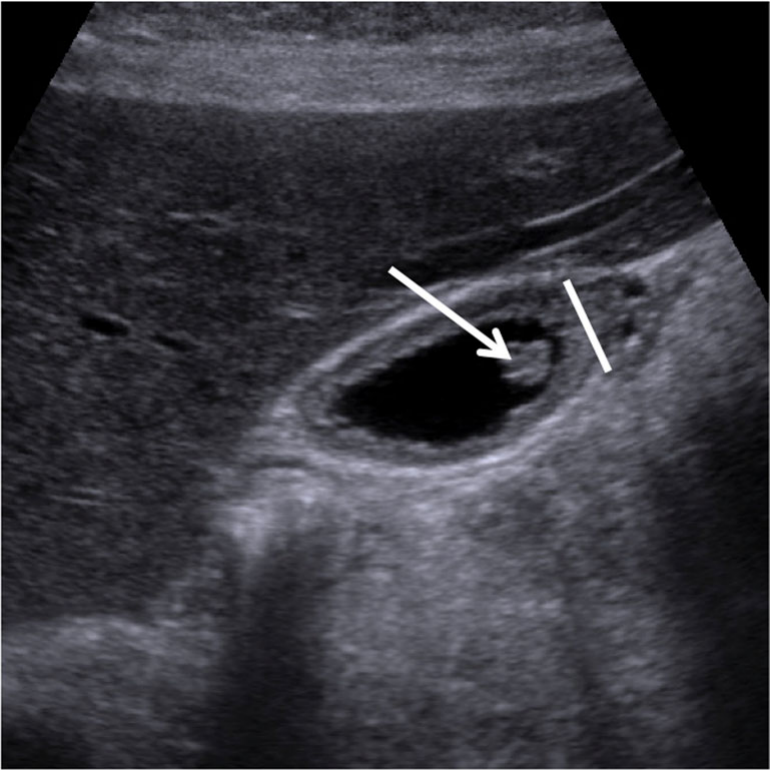
US of gallbladder adenomyomatosis: differential diagnosis with cholesterine polyps. Cholesterine polyps (*arrow*) must not be confused with gallbladder adenomyomatosis (*line*); however, the two alterations may coexist in the same patient

A possible imaging pitfall is the differentiation between *cholesterine polyps* and GA. Polyps appear as solid nodules with exophytic growth inside the gallbladder lumen, whereas GA appears as focal or diffuse mural thickening. Anyway, polyps and adenomyomatosis may coexist in some patients (Fig. 7).

**Fig. 8:**
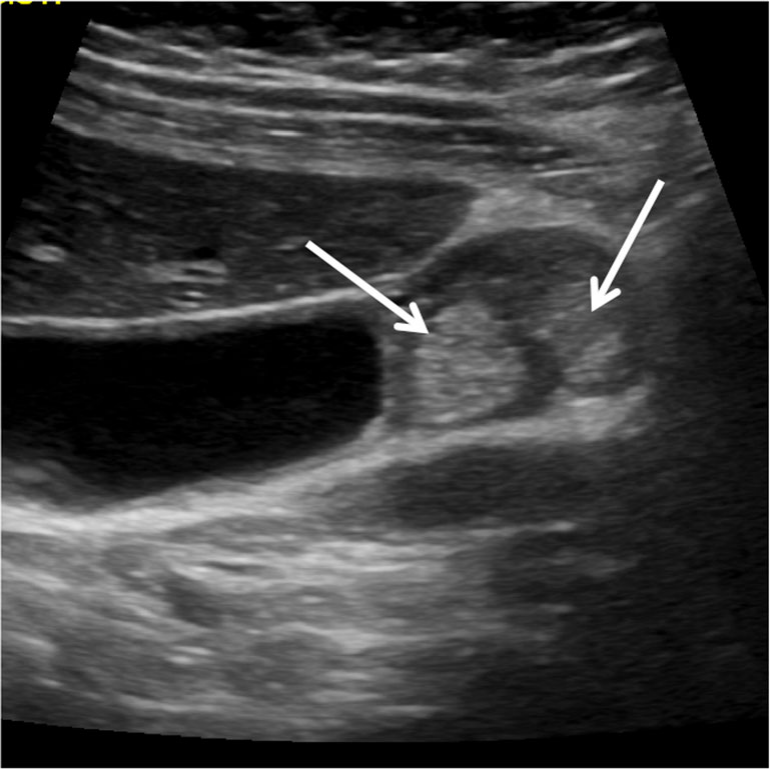
US of gallbladder adenomyomatosis: pitfalls. Cholesterine crystals may accumulate within large Rokitansky–Aschoff sinuses, determining a hyperechoic aspect (*arrows*) without acoustic shadowing

Large round hyperechoic intramural collections without acoustic shadowing or reverberation artefacts, representing cholesterine-filled RAS (Fig. 8), may sometimes be observed. This finding may cause diagnostic doubts.

## Endoscopic ultrasound

Endoscopic ultrasound (EUS) is an invasive imaging modality that is capable of accurately evaluating the gallbladder wall as the high-frequency probe can be positioned in its close proximity without the interposition of other anatomical structures. This results in a higher accuracy in the evaluation of gallbladder wall thickenings in comparison to US. EUS findings are the same as trans-abdominal ones (i.e., gallbladder wall thickening with intramural cystic spaces and/or echogenic foci, comet-tail artefacts and twinkling artefacts) and can be highlighted with higher sensitivity, in particular, in obese patients [24]. The main limitations to EUS reside in its invasiveness, low tolerability and costs; therefore, EUS is not routinely considered for the diagnosis of GA. Moreover, it has been demonstrated that EUS can depict some microcystic spaces that may be present in gallbladder cancer [8].

## Contrast-enhanced ultrasound

Intravenous administration of micro-bubble contrast material represents a useful complement to conventional US and is increasingly used for various indications in abdominal imaging (e.g., for the differential diagnosis of focal liver lesions and for the characterization of renal cysts). In recent years, contrast enhanced ultrasound (CEUS) has been proposed, with encouraging results, also for the differential diagnosis of gallbladder wall thickenings [25, 26] and Tang et al. have demonstrated that contrast material administration significantly increases US sensitivity in the detection of RAS and in the depiction of gallbladder wall continuity in patients with GA [27]. CEUS implicates the use of dedicated low mechanical index presets and intravenous administration of a bolus of 2.4 ml of contrast material, containing 8 μl/ml sulphur hexafluoride microbubbles, followed by a 10-ml saline flush; the target lesion is then scanned for the following 3–5 min in order to assess its vascularization.

### Imaging findings

The thickened gallbladder wall shows the same degree of *enhancement* as the adjacent normal wall in the majority of the cases, whereas a relative hyper-enhancement may be observed in about 15% of the cases. Wall enhancement typically shows a trilaminar pattern during the arterial phase as a consequence of increased mucosal and serosal vascularization. The external layer must show no discontinuities.

**Fig. 9:**
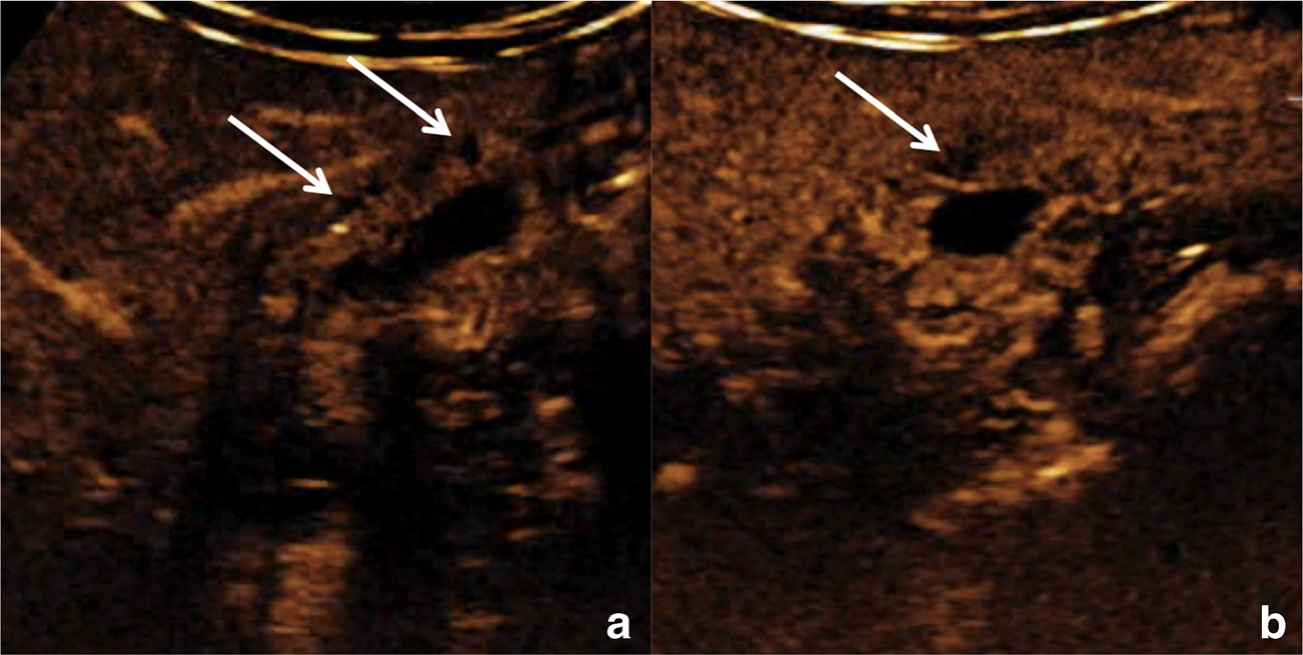
Gallbladder adenomyomatosis: typical contrast-enhanced ultrasound (CEUS) findings. On CEUS, the thickened gallbladder wall shows discrete contrast enhancement, whereas Rokitansky–Aschoff sinuses (*arrows*) appear as avascular structures during every phase of the exam

Avascular spaces, representing *RAS*, must be observed within the thickened gallbladder wall (Fig. 9). RAS appear avascular in every phase of the dynamic study, independently from their content. The identification of avascular spaces within a gallbladder wall thickening is virtually pathognomonic for GA.

### Tips & tricks

Intramural avascular spaces are best appreciated *70*–*100 s* after endovenous contrast material administration when the gallbladder wall shows a homogeneous high-degree enhancement.

### Pitfalls & limitations

The *low mechanical index* preset used for the performance of CEUS may reduce the accuracy in the characterization of gallbladder wall thickenings located in deep positions, away from the abdominal surface and from the probe. This may significantly limit CEUS accuracy in lesions involving the gallbladder neck and in obese patients.

## Magnetic resonance

Thanks to the recent technical developments, to its multiplanarity and to its high tissue contrast resolution, magnetic resonance imaging (MRI) is becoming an increasingly requested imaging technique for the study of gallbladder pathologies [9]. On the other hand, MRI remains a time- and resource-consuming imaging modality and, therefore, the indications for its use must be accurately evaluated. MRI is the imaging modality that offers the highest accuracy in diagnosing GA and, in particular, in differentiating GA from gallbladder carcinoma (accuracy 93.0%) [28].

### Imaging findings

Gallbladder *wall thickening* can be clearly depicted both on T1- and on T2-weighted images, and is not a specific finding. Anyway, MRI warrants high specificity in the diagnosis of GA by accurately ruling out extra-parietal infiltration, which is indicative of gallbladder carcinoma.

**Fig. 10:**
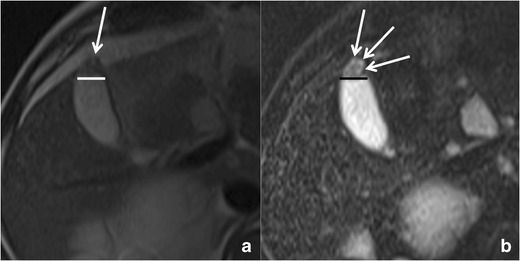
Gallbladder adenomyomatosis: typical MRI findings. On MRI, GA can be identified as a mural thickening (*line*) containing small T2-hyperintense spaces representing RAS (*arrows*). RAS can be better identified on fat-saturated T2-weighted images (**b**) than on non-fat-saturated ones (**a**)

**Fig. 11:**
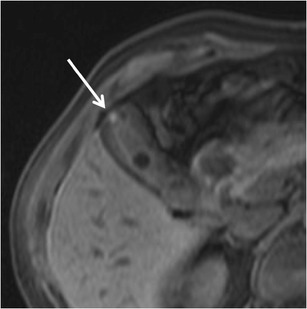
MRI of gallbladder adenomyomatosis: T1-hyperintense Rokitansky–Aschoff sinuses. Rokitansky–Aschoff sinuses (*arrow*) may appear hyperintense on T1-weighted images if containing concentrated bile or calcifications

RAS typically appear markedly hyperintense on T2-weighted images (Fig. 10) [29], hypointense on T1-weighted images and show no contrast enhancement. Anyway, progressive bile concentration and calcification development may change the MRI appearance of RAS that may become increasingly hyperintense on T1-weighted images (Fig. 11) and relatively hypointense on T2-weighted ones.

### Tips & tricks

The use of *fat-saturated* T2-weighted sequences increases RAS conspicuity.

**Fig. 12:**
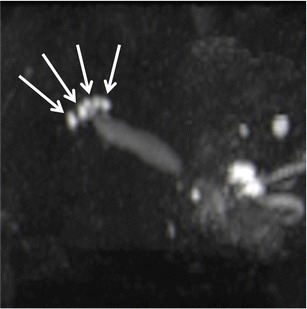
MRI of gallbladder adenomyomatosis: the pearl necklace sign. On heavily T2-weighted images, like in this maximum intensity projection reconstruction of a volumetric MRCP, a lot of RAS can be identified one next to the other around the gallbladder, leading to the so-called pearl necklace sign

MR cholangiopancreatography (MRCP) images can be useful for identifying RAS that sometimes may be overlooked on axial images. On MRCP images, many RAS can be usually observed one next to each other along the involved gallbladder wall leading to the so-called *pearl necklace sign* (Fig. 12) [30].

RAS may be extremely small and, therefore, barely recognizable on thick slab T2-weighted images. The acquisition of *volumetric* respiratory-triggered T2-weighted images increases the sensitivity of MRI in recognizing small RAS and offers the possibility of multiplanar reconstructions.

*Contrast material* administration is not routinely indicated in the suspicion of GA. Indeed, *heavily T2-weighted* fast spin-echo sequences are the most reliable for the identification of RAS.

*Diffusion-weighted imaging* can be helpful in the differential diagnosis between benign and malignant gallbladder wall thickenings. Ogawa et al. [31] demonstrated that malignant thickenings show significantly lower apparent diffusion coefficient (ADC) values than benign ones. Despite this, some overlap exists between benign and malignant lesions.

### Pitfalls & limitations

*T1-hyperintense* RAS can be frequently observed. This must not raise any doubt in the diagnosis of GA, being the consequence of concentrated biliary content, cholesterine crystals or calcifications.

RAS with *different content*, and consequently with different signal intensities, often coexist in the same patient.

## Computed tomography

The finding of a gallbladder wall thickening at computed tomography (CT) may represent a diagnostic dilemma; in fact, unless clear signs of malignancy are present, CT has an unsatisfactory accuracy in their differential diagnosis. The accuracy of CT in differentiating GA from gallbladder carcinoma is between 40 and 75% [32] and a confident diagnosis of GA is possible only if large (at least 3–4 mm) RAS are present.

### Imaging findings

Gallbladder *wall thickening* without extra-parietal growth can be confidently observed at CT, with sensitivity comparable to the other modalities.

**Fig. 13:**
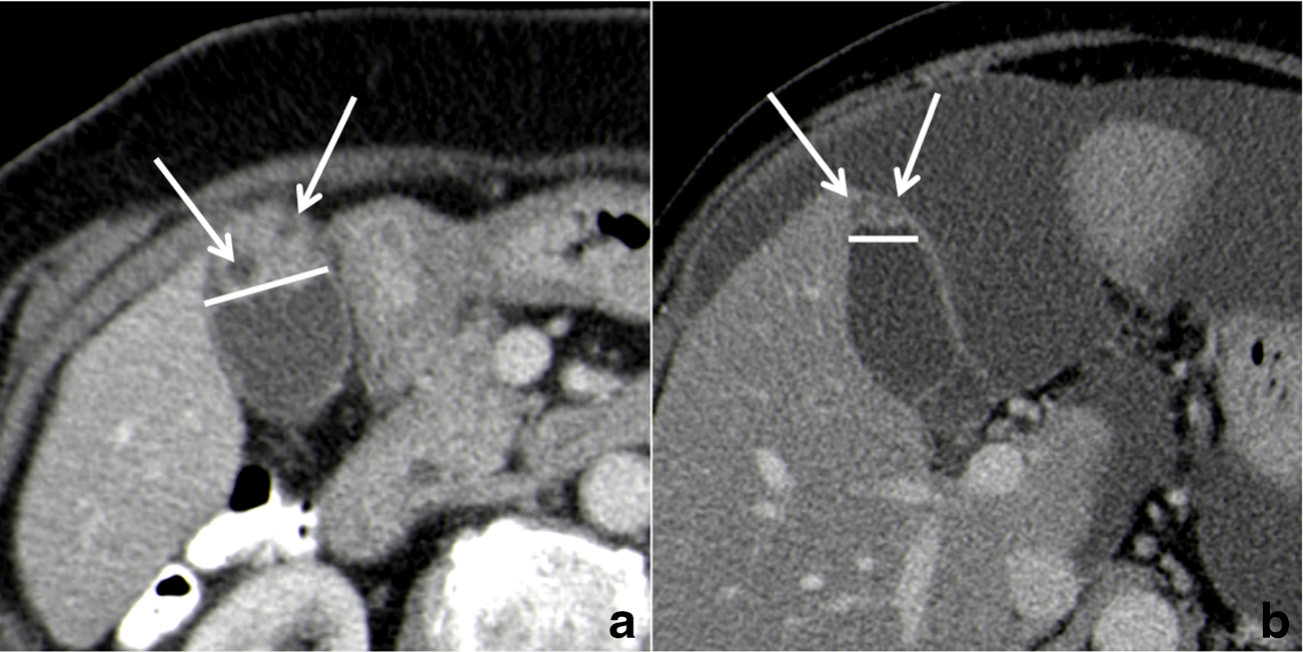
Gallbladder adenomyomatosis: typical CT findings. At CT, gallbladder adenomyomatosis is characterized by mural thickening (*line*) containing cystic spaces representing Rokitansky–Aschoff sinuses (*arrows*). Large RAS can be easily identified on 3-mm-thick reconstructions (**a**), whereas for identifying smaller RAS thin slices evaluation is crucial (**b**)

Well-delimitated *hypodense intramural spaces*, representing RAS, can be confidently recognized only if they reach 3–4 mm in diameter and if they have a clear bile content (Fig. 13). If RAS are clearly identified, CT diagnosis of GA can be made.

**Fig. 14:**
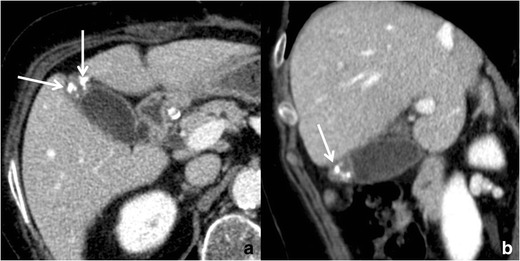
CT of gallbladder adenomyomatosis: intramural calcifications. CT accurately depicts intramural calcifications (*arrows*) that may develop within Rokitansky–Aschoff sinuses and which are pathognomonic for gallbladder adenomyomatosis

Focal *intramural calcifications* (Fig. 14) are virtually pathognomonic for GA. Unfortunately, GA shows intramural calcifications only in a minority of the cases.

### Tips & tricks

CT images evaluation using *thin slice* thickness (1–2 mm) increases accuracy in identifying RAS and in excluding extramural infiltration, although it shows more background noise compared to thicker reconstructions.

Endovenous *contrast material* administration is fundamental in order to increase CT accuracy in RAS recognition and to exclude the presence of extra-parietal growth.

### Pitfalls & limitations

Due to their tiny dimensions and to the low tissue contrast resolution of the technique, RAS cannot be clearly identified at CT in the majority of the cases.

## Positron emission tomography

Despite its low spatial resolution, positron emission tomography (PET) offers the possibility of obtaining metabolic information from body tissues. After i.v. administration of fluorine-18-fluorodeoxiglucose (18 F-FDG), high glucose-consuming tissues (i.e., neoplastic and inflammatory ones) can be identified. PET is not usually performed in the suspicion of GA, but patients affected by GA may sometimes undergo PET for other reasons.

### Imaging findings

**Fig. 15:**
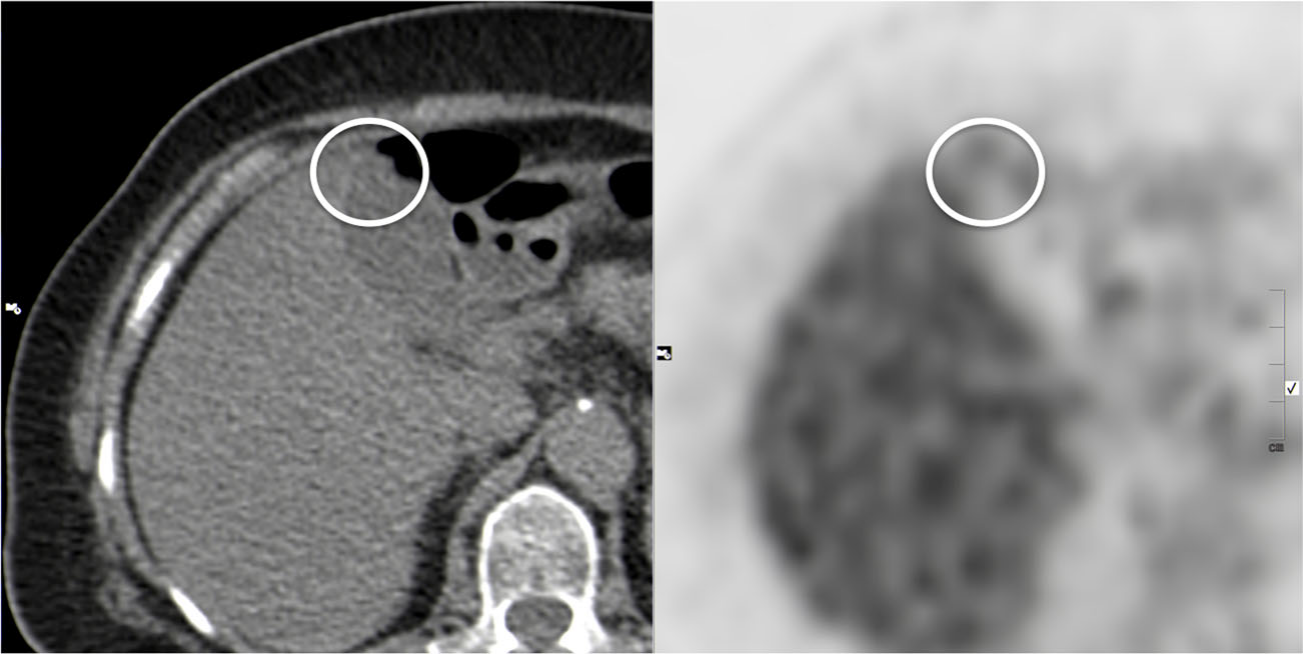
Gallbladder adenomyomatosis: typical PET-CT findings. Gallbladder adenomyomatosis (*circle*) usually shows an F-18FDG uptake equal or lower than the adjacent liver

GA typically shows *no 18 F-FDG uptake* or lower uptake compared to the liver (Fig. 15). This finding is not specific for GA, but may help in excluding malignancy [33].

### Pitfalls & limitations

An acute inflammatory reaction sometimes surrounds RAS, generating an *increased 18 F-FDG uptake* that leads to false positive diagnosis of neoplasm [34].

PET has low spatial resolution and its accuracy in excluding early gallbladder neoplasms may be unsatisfactory in lesions measuring less then 1 cm.

## Conclusions

US represents the imaging modality of choice for diagnosing GA, whereas CEUS should be considered the second-line imaging modality. MRI is the problem-solving technique for unclear cases at US and CEUS. PET may be considered for excluding malignancy in cases undiagnosed at US, CEUS and MRI. CT, endoscopic ultrasound and oral cholecystography are not routinely considered for diagnosing GA.

## Abbreviations

GA: Gallbladder adenomyomatosis
RAS: Rokitansky–Aschoff sinuses
OC: Oral cholecystography
US: Trans-abdominal ultrasound
EUS: Endoscopic ultrasound
CEUS: Contrast-enhanced ultrasound
MRI: Magnetic resonance imaging
ADC: Apparent diffusion coefficient
CT: Computed tomography
PET: Positron emission tomography
